# Two new camaenid land snails (Eupulmonata) from Central China

**DOI:** 10.3897/zookeys.861.35430

**Published:** 2019-07-08

**Authors:** Min Wu, Zheyu Chen, Xiaoran Zhu

**Affiliations:** 1 School of Life Sciences, Nanjing University, Xianlindadao 163, Qixia, Nanjing 210023, China Nanjing University Nanjing China; 2 College of food science and engineering, Wuhan Polytechnic University, Wuhan 430023, China Wuhan Polytechnic University Wuhan China; 3 Hubei Board Nature Technology Service Co., Ltd. Wuhan 430079 China Hubei Board Nature Technology Service Co. Wuhan China

**Keywords:** Bradybaeninae, Camaeninae, Hubei, Sichuan, taxonomy

## Abstract

Two new camaenid land snails are reported from Central China. The new genus, represented by *Sinochloritislii* Wu & Chen, **gen. & sp. nov.**, the type of the genus from Sichuan, is close to *Yakuchloritis* Habe, *Nipponochloritis* Habe, *Neochloritis* Minato and *Trichochloritis* Pilsbry, but is well characterized by the smooth adult shell, highly developed epiphallic papilla, absence of penial caecum, and the presence of an epiphallus-binding muscle that binds the proximal epiphallus to the distal penis. A new species *Bradybaenalinjun* Wu & Chen, **sp. nov.** is described from Hubei Province and is characterized by having two shell bands, a spoon-shaped love dart and the proportionally shortest mucous glands among Chinese congeners.

## Abstract (cn)

摘 要 从华中地区报道了两种坚螺科陆生软体动物的新物种。由新种李氏华砾螺*Sinochloritislii* Wu & Chen, **gen. & sp. nov.** 为属模式种的华砾螺属*Sinochloritis* Wu & Chen, **gen. nov.** 与*Yakuchloritis* Habe, *Nipponochloritis* Habe, *Neochloritis* Minato及毛蜗牛属*Trichochloritis* Pilsbry接近，但因华砾螺属的成体贝壳光滑无毛，成荚器乳突高度发达，具成荚器绑结肌以及交接器盲囊阙如而与上述4属区别。李氏华砾螺记录于四川。另一新种廪君巴蜗牛*Bradybaenalinjun* Wu & Chen **sp. nov.** 记录于湖北，它以两条色带, 呈中空的勺形恋矢及具有相对最短的粘液腺等特征与所有其它中国巴蜗牛属物种相区别。

## Introduction

*Trichochloritis* Pilsbry, 1891 (type species *Helixbreviseta* Pfeiffer, 1862, original designation) was established as a subgenus of *Chloritis* Beck, 1837 to accommodate species that featured a “shell depressed, rather thin, the spire low-convex or plane, last whorl not carinated, but usually obtusely angled around the umbilicus; but little deflexed in front; epidermis not deciduous; apex, as well as the whole shell, hirsute or marked by hair-scars arranged in regular lines. Lip narrowly expanded or reflexed” (Pilsbry 1891). *Trichochloritis* is now recognized as a distinct genus (Schileyko 2007, 2011), which ranges from South China to the Philippines and in Japan (Pilsbry 1891, Azuma 1995, Schileyko 2011). Based on conchological and anatomical features (Azuma 1995), species of the Japanese region that were previously included in *Trichochloritis* have been treated as three separate genera, namely *Yakuchloritis* Habe, 1955, *Neochloritis* Minato, 1982 and *Nipponochloritis* Habe, 1955, and were assigned to the family Bradybaenidae (=Bradybaeninae sensu Bouchet et al. 2017) by Schileyko (2004). Based on the genital morphology of *Trichochloritisbrevidens* (Sowerby, 1841) (Schileyko 2007; Table 1), *Trichochloritis* is unambiguously distinct from the Japanese genera.

Ten Chinese species and subspecies have been assigned to *Trichochloritis* (Table 2). In 1882, Heude described the first species *Helixpercussa* from Wudangshan Mountain, Hubei. Möllendorff (1884) assigned it to *Hadra* Albers, 1850. Pilsbry (1890) placed *H.percussa* in his new genus *Euhadra*. Schmacker and Böttger (1894) followed this arrangement and listed a series of specimens that were considered to be similar to *H.percussa* from Tchen k’eou (=Chengkou, Chongqing), Kaochahien and Patung (=Badong, Hubei). Möllendorff (1884) reported a new hairy-shelled species *Helixhungerfordiana* Nevill, 1884 from Taiwan, with the subspecies *H.hungerfordianarufopila* Möllendorff from Hong Kong and assigned it to ?*Trichia* Hartmann, 1840 (=*Trochulus* Chemnitz, 1786, Hygromiidae). Tryon (1887) followed his arrangement. In 1885, Heude described *H.mola* from Ta-kouan (=Daguan, Zhaotong, Yunnan). In 1887, Gredler described *H.franciscanorum* (Peishan, Hunan), which was assigned to subspecies of *Trichochloritishungerfordiana* by Yen (1939) and was later listed as Chloritis (Trichochloritis) hungerfordianafranciscanorum by Zilch (1974; not “1886” in Zilch 1974). In 1888, Möllendorff described a new species *Helixherziana* from Hoihow (=Haikou, Hainan) and pointed out that it is close to *H.puberula* Heude, 1885, *H.hungerfordiana* and *H.franciscanorum*. Two years later, Heude published *H.molina* from Pa-tong (=Badong, Hubei). In 1891, Pilsbry included *H.herziana*, *H.puberula*, *H.franciscanorum* and *H.hungerfordiana* in his newly established genus *Trichochloritis*. In 1894, Gredler described H. (Fruticicola) adaequata (Secusan, W Hubei), which was assigned to Chloritis (Trichochloritis) by Zilch (1974). Yen assigned *T.submissa* (Deshayes, 1873), *T.diploblepharis* (Möllendorff, 1899), *T.hungerfordiana* (Yen 1939; 1940), *T.mola*, *T.percussa*, *T.herziana*, *T.molina*, *T.hunanensis* Yen, 1939 (Yen 1939), *Helixpatungana* Gredler, 1887 (not “*patungensis*” in Yen 1942; not “Gredler, 1888” in Richardson 1983) and *Helixepixantha* Pfeiffer, 1850 (Yen 1942) to *Trichochloritis*. Among them, *T.submissa* is now the type species of *Trichobradybaena* Wu & Guo, 2003 (Bradybaeninae), and *T.diploblepharis* was assigned to *Plectotropis* Martens, 1860 (subfamily Bradybaeninae sensu Bouchet et al. 2017) by Möllendorff (1899), *H.patungana* was treated by Richardson (1983) as a *Plectotropis* species, and *H.epixantha* was sunk as a synonym as *Bradybaenasimilaris* (Rang, 1831) by Tryon (1887). Chang (1990) moved *Trichochloritishungerfordianus* to *Yakuchloritis* based on genital anatomy.

Until now we have little idea if the species previously placed in *Trichochloritis* form a monophyletic group, although some recent work suggests that *Trichochloritis* as currently understood, consists of species from the Bradybaenidae (=Bradybaeninae sensu Bouchet et al. 2017) and from the Camaenidae (=Camaeninae sensu Bouchet et al. 2017) (Schileyko 2003, 2004, 2007). Here, we report a new species from Sichuan that is conchologically most similar to *T.percussa* but shows marked differences from *Trichochloritis*, *Yakuchloritis*, *Neochloritis* and *Nipponochloritis*. In addition, we describe a new *Bradybaena* species discovered during our recent field work in Hubei Province.

## Methods

Living specimens were relaxed by drowning in water before being transferred to 70% ethanol for fixation, which was replaced with ethanol of the same concentration after three days. The shell and genitalia were measured with digital vernier calipers and from photographs to the nearest 0.1 mm. Whorl number was recorded as described by Kerney and Cameron (1979), with 0.125 whorl accuracy. Soft parts were measured after the specimens were sufficiently fixed in 70% ethanol. Directions used in descriptions: proximal, toward the genital atrium; distal, away from the genital atrium.

Abbreviations: **At** – atrium; **BC** – bursa copulatrix; **BCD** – bursa copulatrix duct; **DS** – dart sac; **DVM** – membranous sac surrounding terminal genitalia; **EBM** – epiphallus-binding muscle, the muscle binding proximal epiphallus to distal end of penis; **Ep** – epiphallus; **EpP** – epiphallic papilla; **Fl** – flagellum; **fma** – fully mature animal; **fms** – empty fully mature shell; **FO** – free oviduct; **HBUMM** – mollusc collection of the Museum of Hebei University, Baoding, China; **MG** – mucous glands; **P** – penis; **PC** – penial caecum; **PR** – penial retractor muscle; **PP** – penial pilaster; **PS** – penis sheath; **Va** – vagina; **VD** – vas deferens.

## Systematics

### Helicoidea Rafinesque, 1815

#### Camaenidae Pilsbry, 1895

##### Bradybaeninae Pilsbry, 1898

###### Sinochloritis

Taxon classificationAnimaliaStylommatophoraCamaenidae

Wu & Chen gen. nov.

http://zoobank.org/926FDE79-21D2-4464-835D-972E04CF300E

####### Type species.

*Sinochloritislii* Wu & Chen, gen. & sp. nov.

####### Diagnosis.

Adult shell smooth. Shell evenly covered with fine granules throughout. Dart sac apparatus absent. Penis sheath absent. Highly developed epiphallic papilla present. Penial caecum absent. Epiphallus-binding muscle connecting proximal epiphallus to distal end of penis. Flagellum present.

####### Description.

Shell depressed. Whorls convex. Suture rather impressed. Protoconch and teleoconch densely and evenly covered with fine granules. Adult shell not hairy or scaly. Peristome abruptly angulated at top; narrowly and uniformly reflexed. Shell glossy; uniformly colored; not banded.

Genitalia. Penis sheath absent. Penis externally simple; internally with several pilasters. Epiphallus internally with a large epiphallic papilla that enters penis; externally with proximal part connected with distal end of penis by strong muscles (epiphallus-binding muscles). Flagellum present. Vas deferens uniformly thin.

####### Etymology.

This new genus is named after “sino” (=China) and “chloritis” (the genus used to include many Chinese *Trichochloritis* species).

####### Distribution.

Sichuan Province.

####### Remarks.

Compared to *Trichochloritis*, *Yakuchloritis*, *Neochloritis* and *Nipponochloritis* (Table 1), the new genus exhibits distinct genital features that justify recognition of a new generic rank. Many Chinese species mentioned above, i.e., the species in *Trichochloritis*, possess general similarity in shell morphology but placement within genera requires evidence from either, or both, reproductive morphology and molecular data.

**Table 1.** Comparison of *Sinochloritis* Wu & Chen, gen. nov. to *Trichochloritis* Pilsbry, 1891 and the other genera previously listed as *Trichochloritis* (Habe 1955, Minato 1982, Azuma 1995, Schileyko 2004, Schileyko 2007, this work). EBM – epihallus-binding muscle, the muscle binding proximal epiphallus to distal end of penis; Ep – epiphallus; EpP – epiphallic papilla; Fl – flagellum; PC – penial caecum; PS – penis sheath.

Groups	Spire	Hair	PS	Ep	EpP	EBM	PC	Fl
*Trichochloritis* Pilsbry, 1891	lower	thin	+	–	–	–	N/A	–
*Yakuchloritis* Habe, 1955	lower	thick	–	+	?	–	–	++
*Nipponochloritis* Habe, 1955	lower	thin	–	+	?	–	+	++/+
*Neochloritis* Minato, 1982	higher	thin	–	+	?	–	–	+
*Sinochloritis* Wu & Chen gen. nov.	higher	N/A	–	+	++	+	–	+

++ developed, + present, –absent, N/A not applicable.

###### Sinochloritis lii

Taxon classificationAnimaliaStylommatophoraCamaenidae

Wu & Chen, gen. & sp. nov.

http://zoobank.org/FCC544B8-3DD5-4785-A1C0-5A2E2240C55E

**Figure 1.**
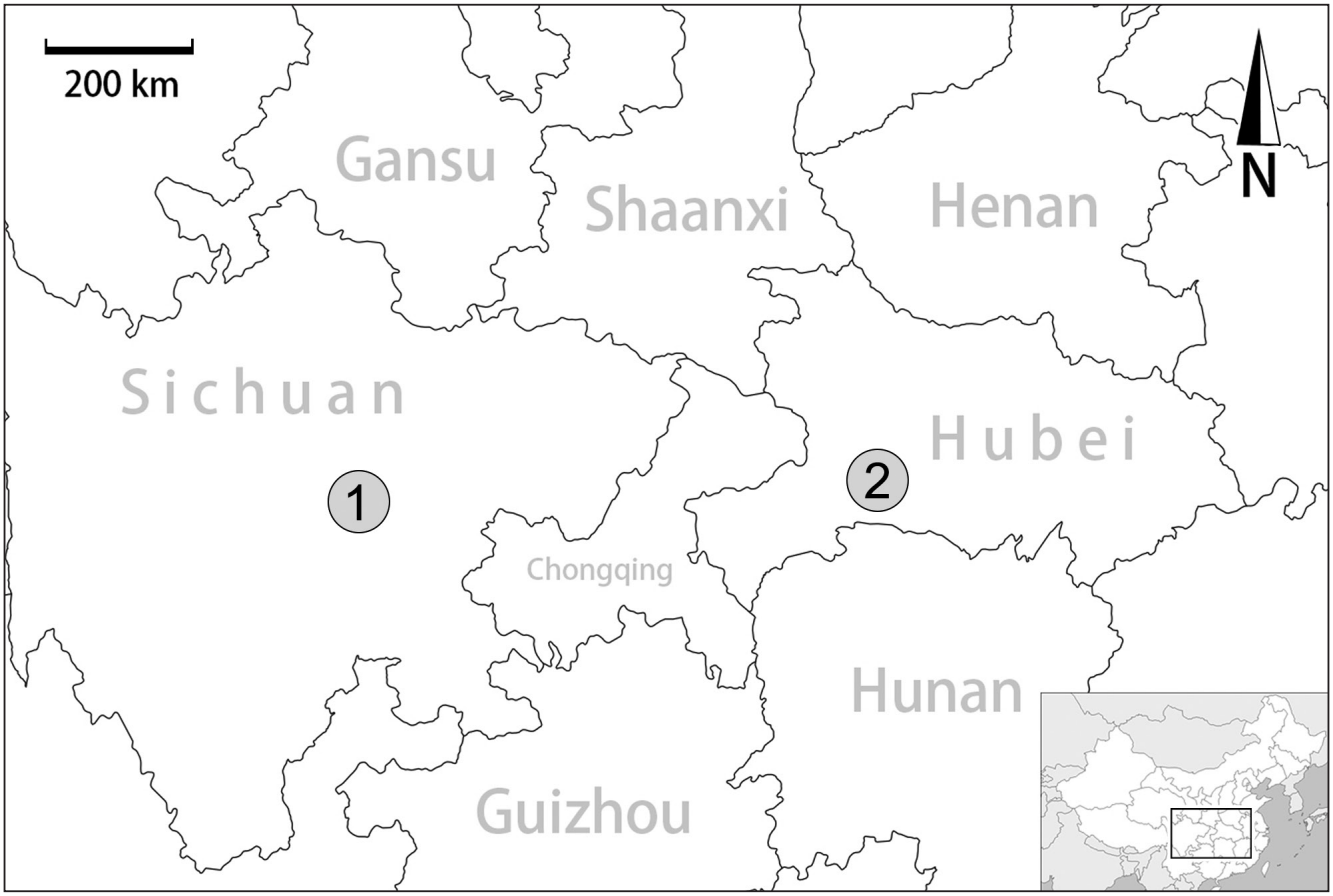
Distribution map. **1***Sinochloritislii* Wu & Chen, gen. & sp. nov.; **2***Bradybaenalinjun* Wu & Chen, sp. nov.

**Figure 2.**
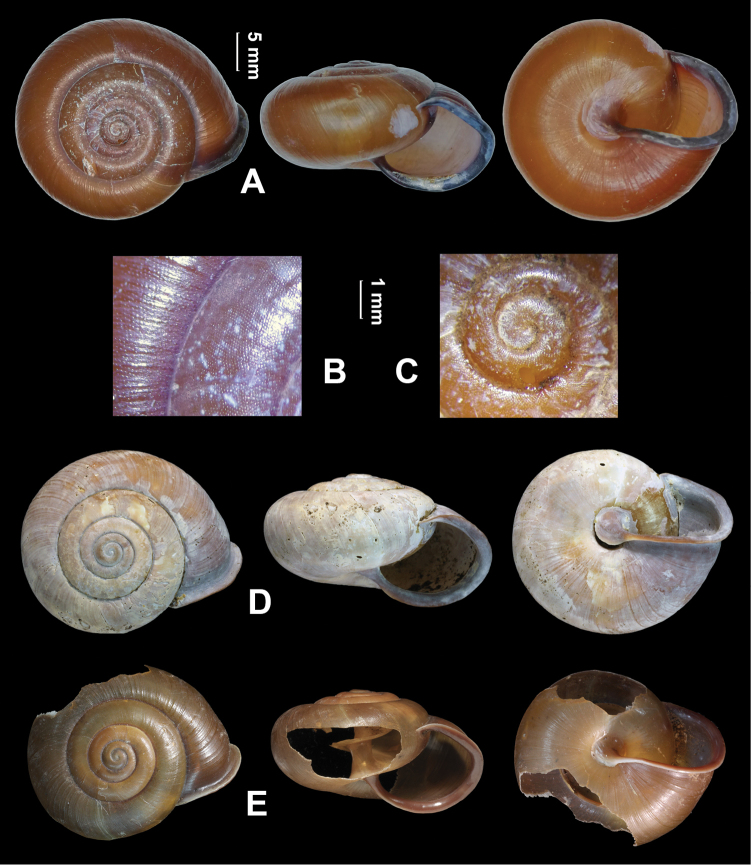
*Sinochloritislii* Wu & Chen, gen. & sp. nov., holotype, HBUMM08294. **A** shell **B** magnified surface of teleoconch **C** magnified embryonic shell **D** paratype HBUMM10008 **E** paratype, HBUMM10009.

**Figure 3.**
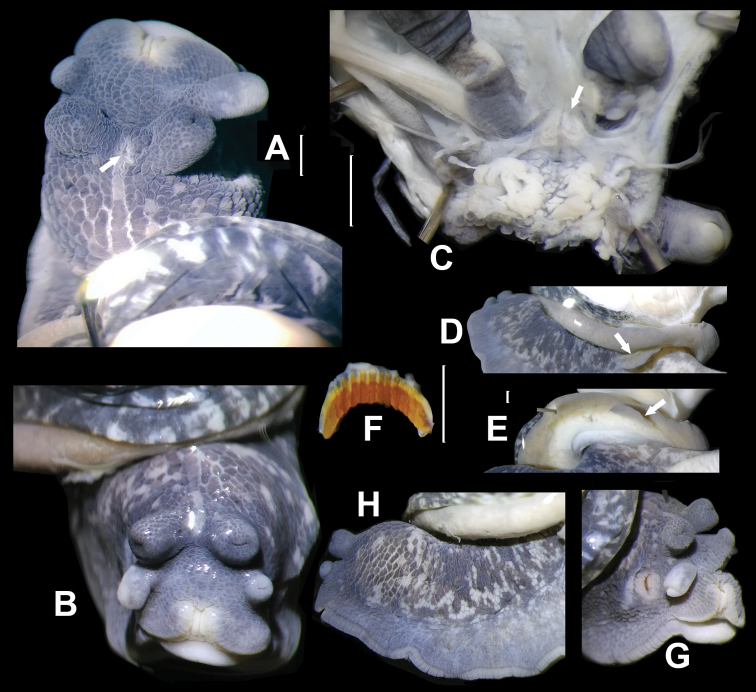
*Sinochloritislii* Wu & Chen, gen. & sp. nov., holotype, HBUMM08294. **A** anterior part of animal, dorsal view, showing the pore (arrowed) of head gland between ommatophore tentacles **B** head of the animal **C** internal body wall of head, showing the head gland (arrowed) between the ommatophore tentacles **D, E** the leaf-shaped appendage (arrowed) on the left margin of mantle, in two views **F** left side of animal, showing coloration and skin pattern **G** right side of head. Scale bars: 1 mm.

**Figure 4.**
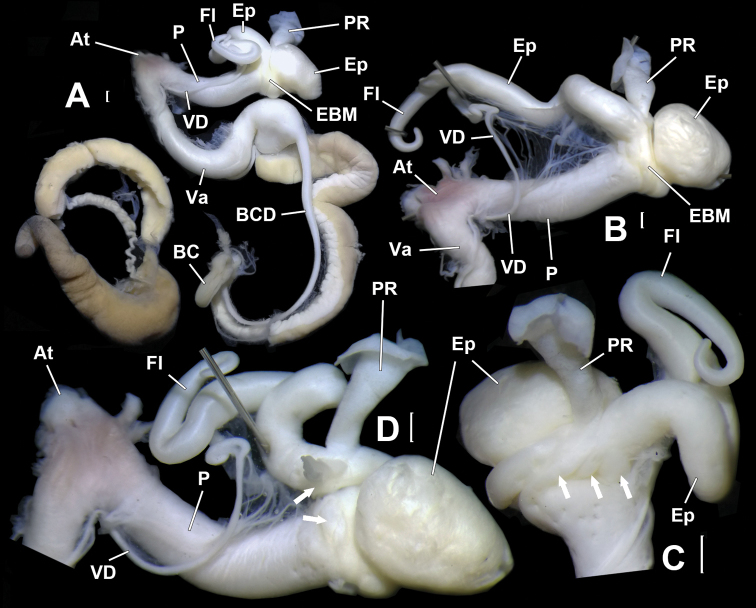
*Sinochloritislii* Wu & Chen, gen. & sp. nov., holotype, HBUMM08294. **A** genitalia, general view, distal part opened **B** male part, intact **C** male part, showing the muscle (arrowed) connecting epiphallus and penis **D** male part, with the muscle connecting epiphallus and penis partially severed (arrowed). Scale bars: 1 mm. At – atrium; BC – bursa copulatrix; BCD – bursa copulatrix duct; EBM – epihallus-binding muscle, the muscle binding the proximal epiphallus to the distal end of penis; Ep – epiphallus; Fl – flagellum; P – penis; PR – penial retractor muscle; Va – vagina; VD – vas deferens.

**Figure 5.**
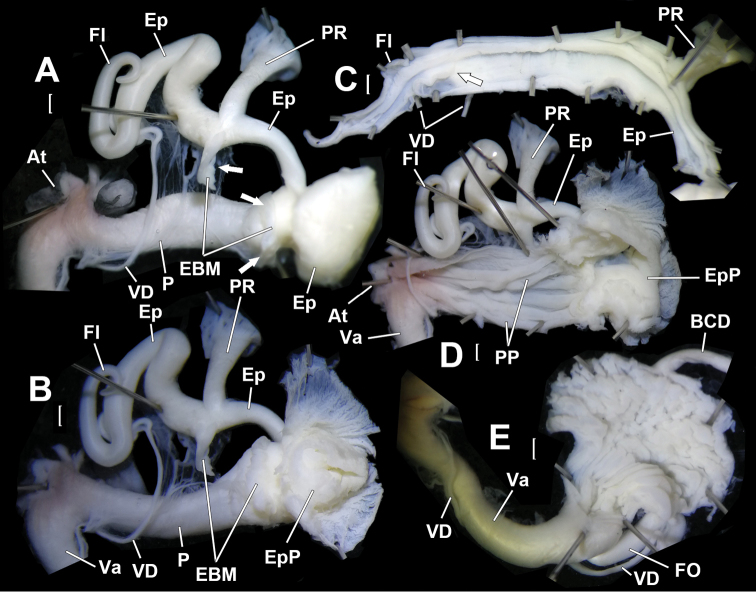
*Sinochloritislii* Wu & Chen, gen. & sp. nov., holotype, HBUMM08294. **A** male part, with the muscle (arrowed) connecting epiphallus and penis completely severed **B** exposed part containing epiphallic papilla **C** exposed epiphallus and flagellum. Arrow indicates insertion of vas deferens **D** opened penis, showing penial pilasters **E** basal part of bursa copulatrix duct, exposed. Scale bars: 1 mm. At – atrium; BCD – bursa copulatrix duct; EBM – epihallus-binding muscle, the muscle binding proximal epiphallus to distal end of penis; Ep – epiphallus; EpP – epiphallic papilla; Fl – flagellum; FO – free oviduct; P – penis; PR – penial retractor muscle; PP – penial pilaster; Va – vagina; VD – vas deferens.

**Figure 6.**
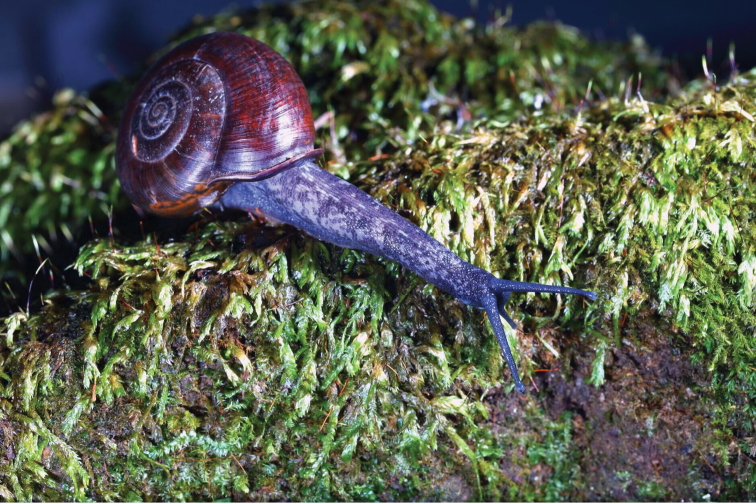
*Sinochloritislii* Wu & Chen, gen. & sp. nov., holotype, HBUMM08294. Habitat, photographer Chenliang Li.

Figs 1 , 2 , 3 , 4 , 5 , 6

####### Type material.

**Holotype**, fully matured animal (HBUMM08294). Sichuan Province, Dujiangyan, Qingchenghoushan, 30°56'39.38"N, 103°28'47.21"E, 1500 m a. s. l., 2018-XI-8, coll. Li, Chenliang & Zhu, Xiaoran. A sample of foot muscle tissue was preserved in 99.7% ethanol at –20 °C (HBUMM08295). **Paratypes**, 1 old fms (HBUMM10008), Sichuan, Dujiangyan, Qinchenghoushan, 1500 m a. s. l., 2018-V, coll. Liu, Zhengping; 1 broken fully matured shell (HBUMM10009), Sichuan, Dujiangyan, Qinchenghoushan, 1500 m a. s. l., 2017-X, coll. Liu, Zhengping.

####### Description.

Shell (Fig. 2). Depressed; thick and solid; dextral. Whorls convex. Suture rather impressed. Umbilicus closed by reflexed columellar lip. Columella oblique. Protoconch and teleoconch densely and uniformly covered with fine granules, without spiral furrows. Aperture oblique; not sinuate at peristome. Body whorl not descending behind aperture. Shell surface without ribs. Growth lines fine. Adult shell not hairy or scaly. Adult body whorl rounded at periphery; basally convex. Ring-like thickening within aperture absent. Peristome thin; abruptly angled at top; narrowly and uniformly reflexed; brownish purple. Callus thin and transparent. Shell glossy; uniformly reddish brown. Measurements (type material): shell height 16.0–17.1 (16.5 ± 0.55) mm, shell breadth 25.0–30.6 (27.0 ± 3.10) mm, aperture height 11.5–12.5 (11.9 ± 0.51) mm, aperture width 13.4–16.8 (14.9 ± 1.72) mm, embryonic shell whorls 1.375–1.500 (1.458 ± 0.072), whorls 4.750–4.875 (4.833 ± 0.072), shell height/ breadth ratio 0.56–0.65 (0.62 ± 0.049).

General anatomy (Fig. 3). A heart-shaped head gland between ommatophore insertions present on inner body wall (Fig. 3C, arrowed), externally with a visible gland pore (Fig. 3A, arrowed). On internal body wall, at the base of ommatophore with two groups of glands each consisted of numerous small sacs (Fig. 3C). On left side of mantle edge, a leaf-shaped appendage present (Fig. 3D, E). Body blueish purple with scattered lighter spots (Fig. 3H). Sole dirty white. Jaw arcuate; with twelve more or less projecting ribs (Fig. 3F).

Genitalia (Figs 4, 5). Penis sheath absent. Penis thick; externally simple; internally with five thick and high plicae/pilasters (Fig. 5D). Epiphallus longer than penis; with section between penial retractor muscle and epiphallic papilla one-third thickness of penis; section between penial retractor muscle and vas deferens insertion much thicker than proximal part but thinner than penis (Fig. 5A); internally with a large peach-shaped epiphallic papilla (approximate size 3.5×4.0×6.0 mm^3^) entering penis (Fig. 5A, B); externally, partially connected with distal penis by strong muscles that insert on penis just opposite to penial retractor muscle (Figs 4C, 4D, 5A, arrowed). Flagellum cylindrical; tapering. Inside flagellum and epiphallus, a long pilaster running from tip of flagellum to epiphallic papilla and a much shorter wavy pilaster running from tip of flagellum to vas deferens insertion (Fig. 5C). Vas deferens thin; of even thickness (Fig. 4A). Vagina subequal to penis in length (Fig. 4A). Base of bursa copulatrix duct expanded and ball-shaped (Fig. 4A); internal wall strongly corrugated (Fig. 5E). Measurement of holotype: P–13.6 mm; Ep–19.3 mm; Fl–8.6 mm; VD–31.4 mm; PR–4.6 mm; Va–18.8 mm; FO–5.4 mm; BC plus BCD–37.9 mm.

####### Etymology.

This species is named in honor of Dr. Chenliang Li who collected and sent us the holotype (HBUMM08294).

####### Distribution.

Sichuan (Qingchengshan), only known from the type locality (Fig. 1).

####### Ecology.

This species was found living in the well-developed forest (Fig. 6).

####### Taxonomic remarks.

The new species has a closed umbilicus, otherwise is very close to *Trichochloritispercussa* in shell size (Table 2), the general shape and the microsculpture of shell. *Camaenahemiclista* (Schmacker & Böttger, 1894) known only from Hubei (Lytschouan=Lichuan), which has a closed umbilicus and is bluntly shouldered (but not visible in Yen 1939: pl. 12, fig. 42) resembles the new species; however, the new species has fewer whorls and a clearly rounded periphery.

**Table 2.** Comparison among *Sinochloritislii* Wu & Chen, gen. & sp. nov. and the Chinese species once placed in *Trichochloritis* Pilsbry, 1891.

	Diameter major (mm)	Height (mm)	Whorls	Hairy	Distribution
*T.adaequata* (Gredler, 1894)	12	7	4^1/2^	No	W Hubei,
*T.herziana* (Möllendorff, 1888)	14.5–17	10.5	5	No	Hainan
*T.hunanensis* Yen, 1939	11	7.2	4^1/2^	No	Hunan
*T.hungerfordiana* (Nevill, 1884)	14.5–18	10.5	5	Yes	Taiwan, Hongkong, Guangdong,
*T.hung.rufopila* (Möllendorff, 1884)	15	9.25	5	Yes	Hongkong
*T.hung.franciscanorum* (Gredler, 1887)	18–22	9–12	5^2/3^–6	No	S Hunan
*T.mola* (Heude, 1885)	30–31	15	4.5 **	No	Yunnan
*T.molina* (Heude, 1890)	14–17	10	4	No	Hubei
*T.percussa* (Heude, 1882) *	26–30	19	5^1/4^	No	Hubei
*T.puberula* (Heude, 1885)	15–18	9	5	Yes	Chongqing
*Sinochloritislii* Wu & Chen, gen. & sp. nov.	25.0–30.6	16.0–17.1	4^3/4^–4^7/8^	No	Sichuan

* The specimens studied by Schmacker and Böttger (1894) were excluded because they are conchologically different forms and might represent species other than *H.percussa*. ** Counted from fig. 5 (Heude 1885: pl. 29).

###### *Bradybaena* Beck, 1837

**Type species**. *Bradybaenasimilaris* (Rang, 1831); original designation.

####### Bradybaena linjun

Taxon classificationAnimaliaStylommatophoraCamaenidae

Wu & Chen sp. nov.

http://zoobank.org/834A1B9F-7272-4188-9FD5-2121A9D26443

**Figure 7.**
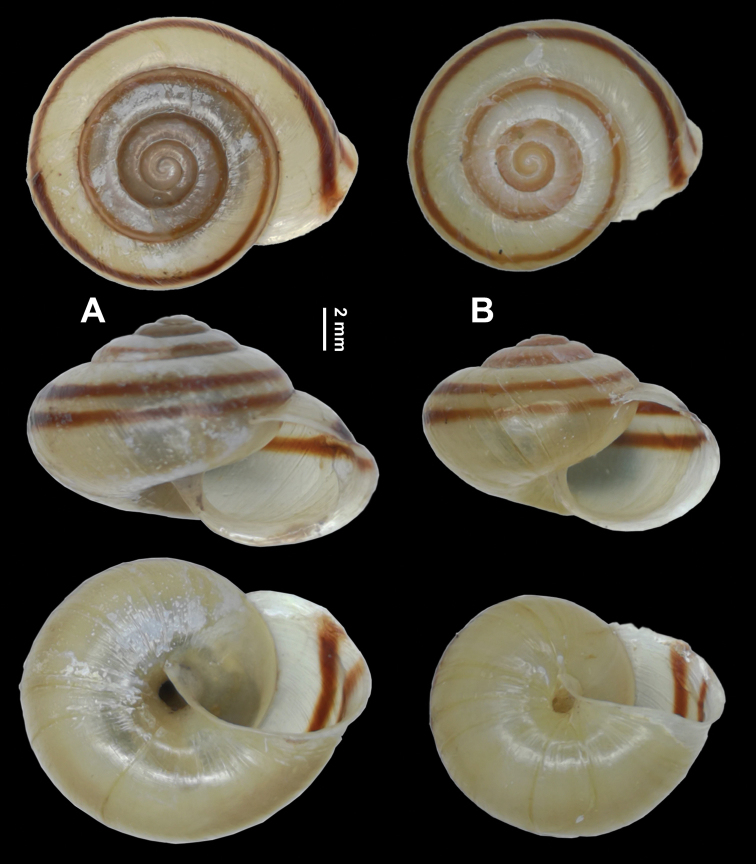
*Bradybaenalinjun* Wu & Chen, sp. nov. **A** holotype, HBUMM08241-specimen 1 **B** paratype, HBUMM08241-specimen 2.

**Figure 8.**
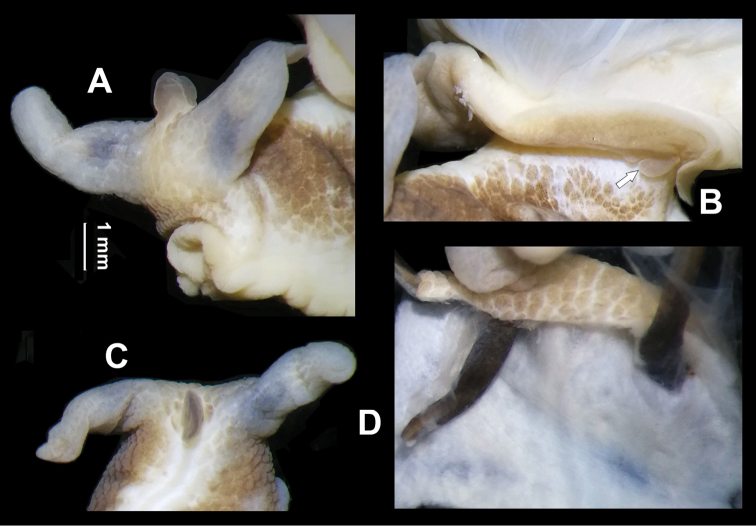
*Bradybaenalinjun* Wu & Chen, sp. nov., holotype, HBUMM08241-specimen 1. **A, C** showing head wart **B** leaf-shaped appendage (arrowed) on left margin of mantle **D** internal body wall between ommatophore insertions, showing no gland.

**Figure 9.**
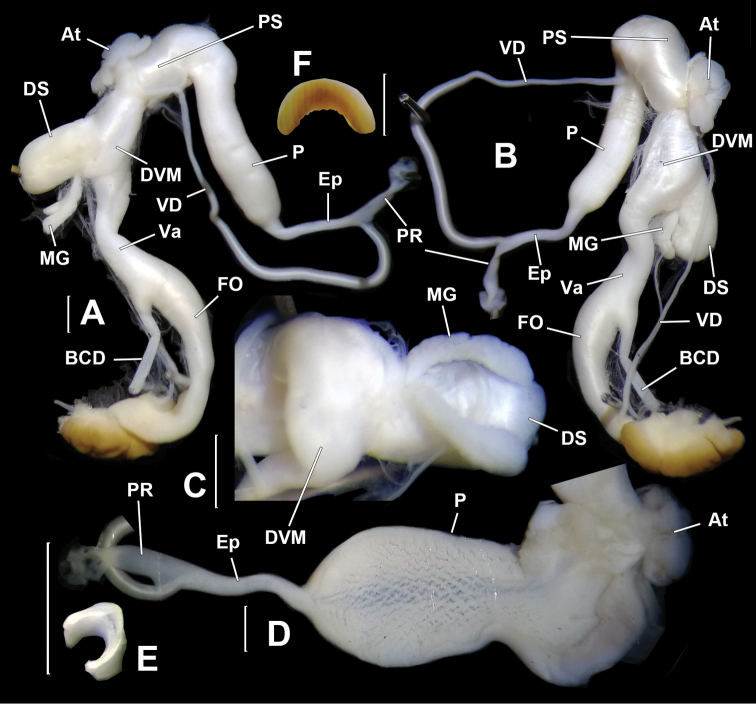
*Bradybaenalinjun* Wu & Chen, sp. nov., holotype, HBUMM08241-specimen 1. **A, B** both sides of genitalia, general view **C** bottom of dart apparatus, showing mucous glands insertion **D** penis, exposed **E** love dart in cross section **F** jaw. Scale bars: 1 mm. At – atrium; BCD – bursa copulatrix duct; DS – dart sac; DVM – membranous sac surrounding terminal genitalia; Ep – epiphallus; FO – free oviduct; MG – mucous glands; P – penis; PR – penial retractor muscle; PS – penis sheath; Va – vagina; VD – vas deferens.

**Figure 10.**
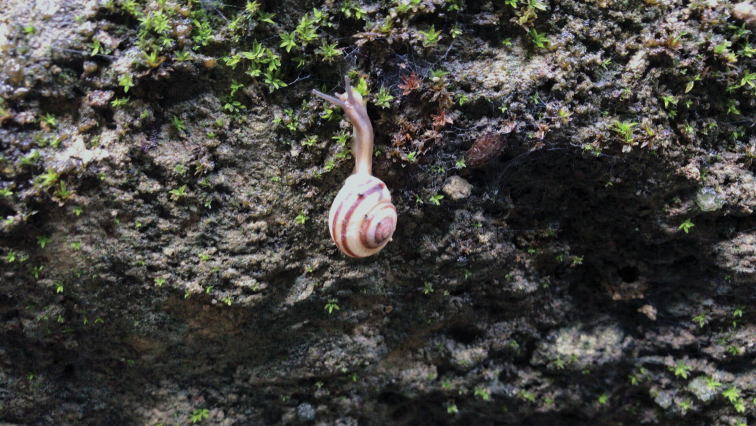
*Bradybaenalinjun* Wu & Chen, sp. nov., holotype, HBUMM08241-specimen 1. Habitat, photographer Zheyu Chen.

Figs 1 , 7 , 8 , 9 , 10

######## Material examined.

**Holotype**, fma (HBUMM08241-specimen 1, Fig. 7A). Hubei Province, Yichang, Changyang Tujia Autonomous Prefecture, Longzhoupin; 31°28'9"N, 111°11'14"E, 103 m a. s. l.; 2018-VII; coll. Chen, Zheyu. **Paratype**, 1 fma (HBUMM08241-specimen 2, Fig. 7B), the same collection information as holotype. Foot muscle was cut off and preserved in 99.7% alcohol at –20 °C (HBUMM08242).

######## Diagnosis.

Shell depressed; dextral. Columella oblique. Periphery rounded. A peripheral and a supraperipheral chestnut band present. Penis internally with numerous crossing pilasters of equal thickness that form a network. Love dart hollow and C-shaped in cross section. Accessory sac externally invisible. Mucous glands two, very short thyrsiform (not branched) tubes; entering accessory sac through simple pore. Shell about 4.5 whorls, breadth 13–17 mm.

######## Description.

Shell (Fig. 7). Depressed; thin; dextral. Whorls convex. Suture impressed. Half umbilicus covered by reflexed columellar lip. Columella oblique. Protoconch not granulate, smooth. Teleoconch with dense spiral furrows. Aperture oblique; not sinuate at peristome. Body whorl slightly descending behind aperture. Shell surface without ribs. Growth lines fine. Adult shell not hairy or scaly. Adult body whorl rounded at periphery; basally convex. Ring-like thickening within aperture absent. Peristome thin; slightly reflexed. Callus thin and transparent. Shell glossy; uniformly brownish yellow; with a peripheral and a supraperipheral chestnut bands. Measurements (holotype is larger in size): shell height 8.8–10.7 mm, shell breadth 13.2–16.6 mm, aperture height 5.9–6.0 mm, aperture width 7.0–9.7 mm, embryonic shell whorls 1.625, whorls 4.250–4.625, shell height/ breadth ratio 0.64–0.67.

General anatomy (Figs 8, 9F). A high head wart between ommatophores present (Fig. 8A, C). On corresponding internal body wall no particular structure present (Fig. 8D). On left side of mantle edge, a leaf-shaped appendage present (Fig. 8B, arrowed). Body light brown; with whitish striae posterior to wart. Sole creamy white. Jaw arcuate; with about thirteen more or less projecting ribs (Fig. 9F).

Genitalia (Fig. 9). Membranous sac surrounding terminal genitalia present (Fig. 9A, B). Penis sheath about 1/3 penis length. Penis very thick; externally simple. Penial retractor muscle inserting on epiphallus. Epiphallus slightly thicker than vas deferens. Flagellum absent. Epiphallic papilla absent (Fig. 9D). Penis internally with numerous crossing pilasters of equal thickness that form a network (Fig. 9D). Dart sac present. Love dart spoon-shaped, hollow and C-shaped in cross section (observed in holotype, Fig. 9E). Accessory sac invisible externally (Fig. 9C). Poly-layered structure present between mucous gland insertion and vagina. Mucous glands two tubes; much shorter than dart sac in length; each thyrsiform rather than branched (Fig. 9A, C); entering accessory sac through simple pore. Vagina about half of penis in length. Measurement of holotype: DS–4.6 mm long, 1.4 mm broad; MG–1.7 mm; PS–1.2 mm; P–7.1 mm; Ep–2.6 mm; VD–21.5 mm; PR–1.7 mm; Va–4.5 mm; FO–2.8 mm.

######## Etymology.

The new species is named after the legendary tribal leader “Lin-Jun (廪君)” of the Tujiazu people who live at the type locality.

######## Distribution.

Hubei (Changyang), only known from the type locality.

######## Ecology.

This species was found living in a well-established secondary forest, on limestone cliffs, often in cracks (Fig. 10). A large number of broken shells, presumably caused by bird predation, were observed at the type locality.

######## Taxonomic remarks.

The new species is assigned to *Bradybaena* because of the presence of a smooth protoconch, membranous sac surrounding terminal genitalia, poly-layered structure in dart apparatus, two mucous glands and the absence of a flagellum; characters that are consistent with the type of the genus *B.similaris* (Wu 2004).

On the left side of the mantle edge, this species possesses a leaf-shaped appendage (Fig. 8B). The existence of this structure in other bradybaenine genera is not known except in *Sinochloritislii* Wu & Chen, gen. & sp. nov. described here (Fig. 3D, E). In our other work on *Bradybaena* this structure is observed (*Bradybaena* sp., HBUMM06125, Wuyuan, Jiangxi Province, 147 m, 29°22'18.8"N, 118°02'45.2"E, 2007-V-26; unpublished data).

Only a few Chinese species in the subfamily Bradybaeninae have double bands. The double-banded shells occur more frequently in *Cathaica* Möllendorff, 1884 than in *Bradybaena* where only four species exhibit double bands, namely *B.billiana* (Heude, 1882), *B.mimicula* (Heude, 1888), *B.diplodesma* (Möllendorff, 1899), *B.sueshanensis* Pilsbry, 1934 (Heude 1882, 1888; Möllendorff 1899; Pilsbry 1934). Although the new species has double bands, in aspect of shell morphology it most resembles *B.qixiaensis* Wu & Asami, 2017. However, the new species has very short mucous glands which are proportionally the shortest in the subfamily Bradybaeninae, the thyrsiform mucous gland duct, and the spoon-shaped love dart, which distinguish this species from all Chinese *Bradybaena* species with known genital anatomy.

## Supplementary Material

XML Treatment for Sinochloritis

XML Treatment for Sinochloritis lii

XML Treatment for Bradybaena linjun

## References

[B1] Azuma M (1995) Colored Illustrations of the Land Snails of Japan. Hoikusha, Osaka, 343 pp. [80 pls.]

[B2] Bachmann O Gredler V (1894) Zur Conchylien-Fauna von China. XVIII. Stück. Annalen des K. K. Naturhistorischen Hofmuseums 9: 415–429.

[B3] Bouchet P Rocroi J-P Hausdorf B Kaim A Kano Y Nützel A Parkhaev P Schrödl M Strong EE (2017) Revised classification, nomenclator and typification of gastropod and monoplacophoran families. Malacologia 61(1–2): 1–526. 10.4002/040.061.0201

[B4] Chang KM (1990) Systematics on *Trichochloritishungerfordianus* from Taiwan. Bulletin of Malacology ROC 15: 35–41.

[B5] Gredler V (1887) Zur Conchylien-Fauna von China. IX. Stück. Malakozoologische Blätter, 1–20.

[B6] Habe T (1955) Anatomical studies on Japanese land snails (3). Venus 18(4): 221–234.

[B7] Heude PM (1882–1890) Notes sur les mollusques terrestres de la vallée du Fleuve Bleu. Mémoires concernant l’histoire naturelle de l’Empire chinois 1882: 1–84; 1885: 89–132; 1890: 125–188.

[B8] Heude PM (1888) Diagnoses Molluscorum novorum, in Sinis collectorum (3). Journal de Conchyliologie 36: 235–243.

[B9] Kerney MP Cameron RAD (1979) A Field Guide to the Land Snails of Britain and North-West Europe. Collins, London, 288 pp. [24 pls.]

[B10] Minato H (1982) Land shell fauna of Ujigunto and Kusakakigunto Islets, the Southwestern Kyushu, Japan, with the descriptions of a new genus and six new species. Venus 41(2): 124–140.

[B11] Möllendorff OF (1884) Materialien zur Fauna von China. Jahrbücher der Deutschen Malakozoologischen Gesellschaft 11: 307–390. [pls 7–9.]

[B12] Möllendorff OF (1888) Diagnoses specicrum novarum sinensium. Nachrichtsblatt der Deutschen Malakozoologischen Gesellschaft, 38–44.

[B13] Möllendorff OF (1899) Binnen-Mollusken aus Westchina und Centralasien. I. Annuaire du Musée Zoologique de l’Académie Impériale des St.-Petersburg 4: 46–144. [pls 2–8.] 10.5962/bhl.title.13125

[B14] Pilsbry HA (1890–1891) In: Tryon GW, Pilsbry HA (Eds) Manual of Conchology (2)6: 5–324. [pls 1, 39–69.]

[B15] Pilsbry HA (1934) Zoological results of the Dolan West China expedition of 1931, Part II, Mollusks. Proceeding of the Academy of natural Sciences of Philadelphia 86: 5–28. [6 pls]

[B16] Richardson L (1983) Bradybaenidae: Catalog of species. Miscellaneous Publications of the Department of Malacology of the Academy of Natural Sciences of Philadelphia 9: 1–207.

[B17] Schileyko AA (2003) Treatise on recent terrestrial pulmonate molluscs. Part 11. Trigonochlamydidae, Papillodermidae, Vitrinidae, Limacidae, Bielziidae, Agriolimacidae, Boettgerillidae, Camaenidae. Ruthenica Supplement 2: 1467–1626.

[B18] Schileyko AA (2004) Treatise on recent terrestrial pulmonate molluscs. Part 12. Bradybaenidae, Xanthonychidae, Epiphragmophoridae, Helminthoglyptidae, Elonidae, Sphincterochilidae, Cochlicellidae. Ruthenica Supplement 2: 1627–1763.

[B19] Schileyko AA (2007) Treatise on recent terrestrial pulmonate molluscs. Part 15. Oopeltidae, Anadenidae, Arionidae, Philomycidae, Succineidae, Athoracophoridae. Additions and corrections. Indexes. Ruthenica Supplement 2: 2049–2209.

[B20] Schileyko AA (2011) Check-list of land pulmonate molluscs of Vietnam (Gastropoda: Stylommatophora). Ruthenica 21(1): 1–68.

[B21] Schmacker B Böttger O (1894) Description of some Chinese land-shells. Proceedings of the Malacologial Society 1: 169–174. 10.1093/oxfordjournals.mollus.a064109

[B22] Tryon GW (1887) Manual of Conchology (2)3: 1–313. [pls 1–63.]

[B23] Wu M Guo J (2003) Contribution to the knowledge of Chinese terrestrial malacofauna (Helicoidea): description of a new bradybaenid genus with three species. The Veliger 46(3): 239–251.

[B24] Wu M (2004) Preliminary phylogenetic study of Bradybaenidae (Gastropoda: Stylommatophora: Helicoidea). Malacologia 46(1): 79–125.

[B25] Wu M Asami T (2017) Taxonomical notes on Chinese camaenids with description of three species (Gastropoda: Pulmonata). Molluscan Research, 10.1080/13235818.2017.1380145

[B26] Yen TC (1939) Die chinesischen Land- und Süßwasser-Gastropoden des Natur-Museums Senckenberg. Abhandlungen der Senckenbergischen Naturforschenden Gesellschaft 444: 1–234. [16 pls.]

[B27] Yen TC (1942) A Review of Chinese Gastropods in the British Museum. Proceedings of the Malacological Society of London 24: 170–288. [pls 11–28.]

[B28] Zilch A (1974) Vinzenz Gredler und die Erforschung der Weichtiere Chinas durch Franziskaner aus Tirol. Archiv für Molluskenkunde 104(4/6): 171–228.

